# Cystic lung tuberculosis in children: A series of five cases

**DOI:** 10.4102/sajr.v27i1.2725

**Published:** 2023-09-28

**Authors:** Chetna Mishra, Pooja Abbey, Rama Anand, Varinder Singh, Ravinder Kaur

**Affiliations:** 1 Department of Radiology, Lady Hardinge Medical College, New Delhi, India; 2 Department of Pediatrics, Lady Hardinge Medical College, New Delhi, India; 3 Department of Microbiology, Lady Hardinge Medical College, New Delhi, India

**Keywords:** pulmonary tuberculosis in children, cystic lung disease, pneumothorax, pulmonary tuberculosis, TB

## Abstract

Frequent imaging manifestations of pulmonary tuberculosis are airspace or interstitial nodules with or without tree-in-bud nodules, consolidation, cavitation, ground glass opacity, miliary nodules, lymphadenopathy and pleural effusion. It is unusual to encounter cystic changes in patients with pulmonary tuberculosis, and these findings should be differentiated from other cystic lung diseases. This case series describes five cases of cystic lung disease in children with tuberculosis (TB) with illustrative chest radiography and CT findings.

**Contribution:**

The manuscript highlights the need to consider tuberculosis as a possible cause of acquired cystic lung disease in appropriate clinical settings, particularly in endemic regions.

## Introduction

The diagnosis and management of pulmonary tuberculosis (PTB) in children remains challenging because the paucibacillary nature of the disease in children and immunocompromised patients makes microbiological confirmation difficult. Chest imaging plays a key role by suggesting the tuberculous aetiology for early diagnosis and treatment in children with PTB.

Pulmonary tuberculosis is classified as primary or post-primary (reactivation) TB based on age, underlying immune status and prior exposure. Infants and children are frequently affected by primary TB, which is characterised by lymphadenopathy with concomitant parenchymal abnormalities such as consolidation and pleural effusion without loculation. Post-primary (reactivation) TB is caused by re-infection or reactivation of dormant bacilli from primary infection in previously sensitised individuals.^1^ Post-primary (reactivation) TB primarily affects adolescents and adults and the usual imaging manifestations include airspace or interstitial nodules with or without a tree-in-bud (TIB) appearance, consolidation, cavitation and empyema.^2^

It is unusual to encounter cystic changes in patients with PTB. Lung cysts are well-defined, thin-walled, epithelial lined airspaces within the lung parenchyma and may arise from a variety of causes.^1^ Pulmonary tuberculosis with cystic changes needs to be differentiated from other cystic lung diseases and mimics, such as pneumatocoele, cavity, bronchiectasis and honeycombing. Early diagnosis enables appropriate management and reduces long-term morbidity and mortality.

This case series illustrates five cases of cystic lung changes in children with TB in the absence of human immunodeficiency virus (HIV) infection. The findings at chest radiography (CXR) and CT are presented.

## Case series

### Case 1

**FIGURE 1:**
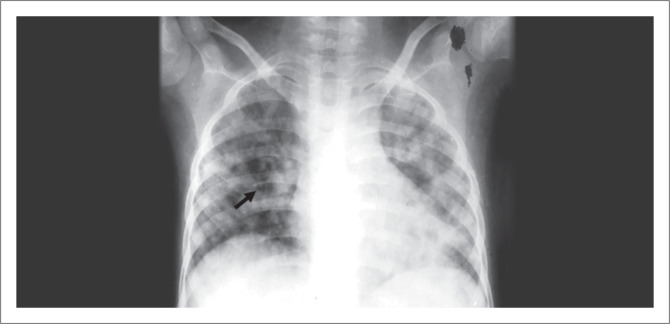
Chest radiograph of a 12-year-old female, shows bilateral air space opacities with few cystic lucencies in the right perihilar region (black arrow).

**FIGURE 2:**
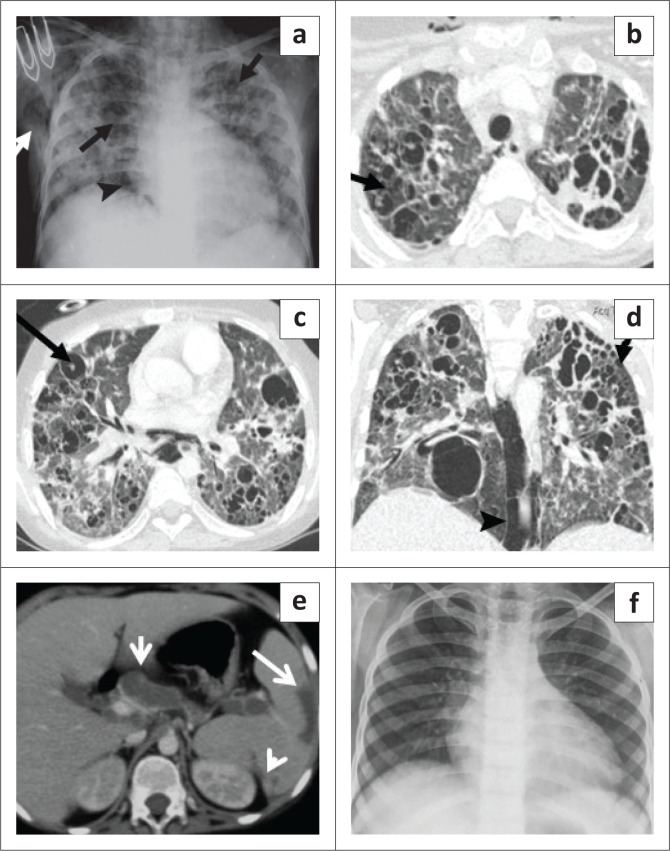
Chest radiography (CXR) (a) shows bilateral patchy lung opacities with multiple cystic lucencies (black arrows), subcutaneous emphysema (white arrow) and pneumomediastinum (arrowhead). Contrast-enhanced CT chest axial (b, c) and coronal (d) images reveal multiple cysts of varying sizes in both lungs (short arrow), pulmonary interstitial emphysema (long arrow) and pneumomediastinum (arrowhead). (e) Axial section through the abdomen shows necrotic periportal lymph nodes (short arrow), splenic infarct (long arrow) and splenic granulomas (arrowhead). (f) Follow-up CXR (after 5 months) appeared normal.

A 12-year-old girl, on treatment for autoimmune haemolytic anaemia, presented with a history of intermittent fever for 1-month, non-productive cough for 3 weeks and shortness of breath over the preceding 7 days. On examination, the patient was febrile with a heart rate (HR) of 156 beats/min and a high respiratory rate (RR) of 64/min. Chest radiography demonstrated ill-defined, bilateral air space opacities, with a few cystic lucencies in the right perihilar region (Figure 1). GeneXpert (sputum) detected mycobacterium tuberculosis (MTB) and the patient was started on antitubercular therapy (ATT). The respiratory failure necessitated ventilator support. Twenty days post-admission, the patient experienced sudden respiratory distress and a repeat CXR (Figure 2) demonstrated patchy opacities with an increased number of cystic lucencies in both lungs, along with the development of subcutaneous emphysema and pneumomediastinum. Contrast-enhanced CT (CECT) (Figure 2) revealed multiple thin-walled cysts, ground glass opacities (GGOs) and a few nodules bilaterally, along with pulmonary interstitial emphysema and pneumomediastinum. Multiple hypodense splenic granulomas and a few enlarged mediastinal and periportal lymph nodes were also seen. The patient responded well to treatment and was weaned off the ventilator within 10 days. A follow-up CXR (Figure 2) 5 months later appeared normal.

### Case 2

**FIGURE 3:**
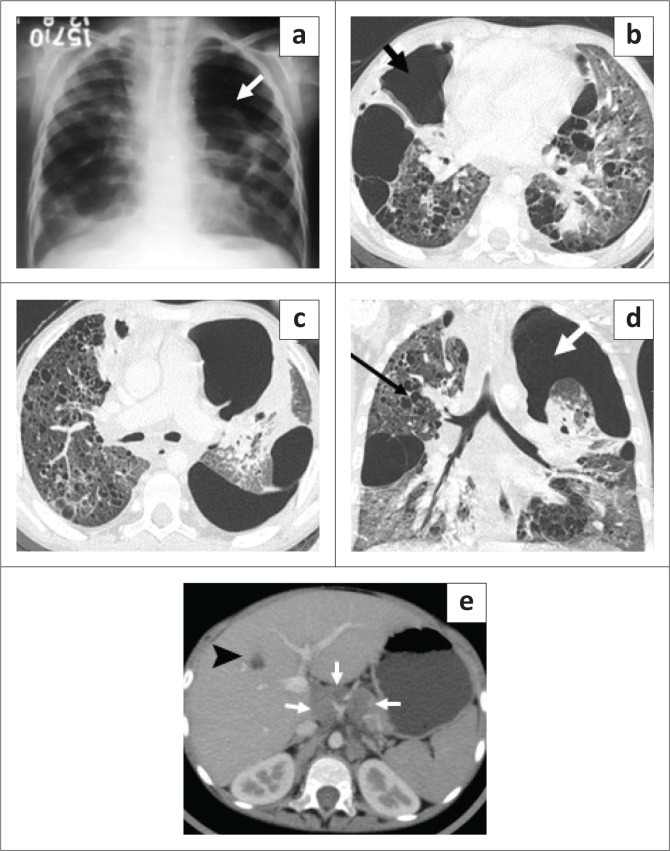
A 7-year-old boy with disseminated TB on antitubercular therapy. Chest radiography (CXR) (a) shows a loculated left pneumothorax (arrow) and cystic lucencies in both lungs. Contrast-enhanced CT chest axial (b, c) and coronal (d) images show a left pneumothorax (white arrow), cavitation (short black arrow) and innumerable variable-sized cysts on both lungs (long arrow) with a few ground glass opacities. Axial section of the abdomen (e) revealed multiple enlarged periportal, paraaortic and coeliac lymph nodes (arrows) and a hepatic granuloma (arrowhead).

**FIGURE 4:**
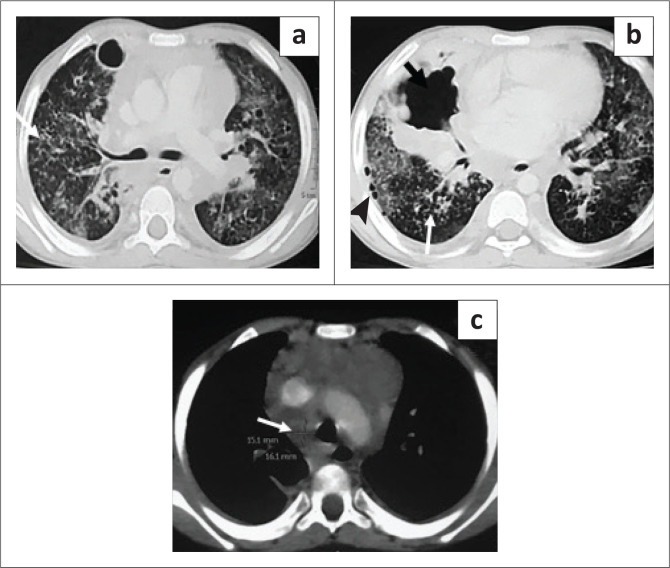
Axial CT performed 2 months prior, (a, b) showed cavitation in the right middle lobe (black arrow) with ground glass opacities in both lungs, a few tiny cysts in the right lung (arrowhead) and multiple bilateral nodules (white arrow). Axial mediastinal window (c) shows enlarged right paratracheal lymph nodes (arrow).

A 7-year-old boy, previously diagnosed with disseminated TB, on ATT for 3 months and on home oxygen therapy, presented to the paediatric outpatient department with complaints of intermittent increased cough from before and was found to have decreased oxygen saturation on room air. He underwent a CXR (Figure 3) that revealed a left loculated pneumothorax. The patient was admitted for assessment of multidrug resistant (MDR) TB and air leak. Contrast-enhanced CT chest (Figure 3) confirmed the left pneumothorax and revealed a thick-walled cavity in the right middle lobe and multiple cysts in both lungs. A previous CECT chest 2 months prior (Figure 4) was reviewed and revealed GGOs, a few tiny cysts, multiple bilateral nodules, along with necrotic mediastinal and abdominal lymphadenopathy, and a few small hypodense lesions in the liver suggestive of non-calcified granulomas. There was an interval increase in the number and size of the lung cysts, with a decrease in the number of nodules. Sputum microscopy and culture were negative. Bronchoscopy indicated signs of chronic inflammation and destruction of the distal bronchial segments and provided abnormal early entry into numerous cavitary structures, unlike the non-diseased areas where the distal branches gradually became smaller in size and did not allow entry into the lung parenchyma. Bronchial washings sent for culture were positive for TB (rifampicin sensitive). The patient was continued on ATT and demonstrated subsequent improvement.

### Case 3

**FIGURE 5:**
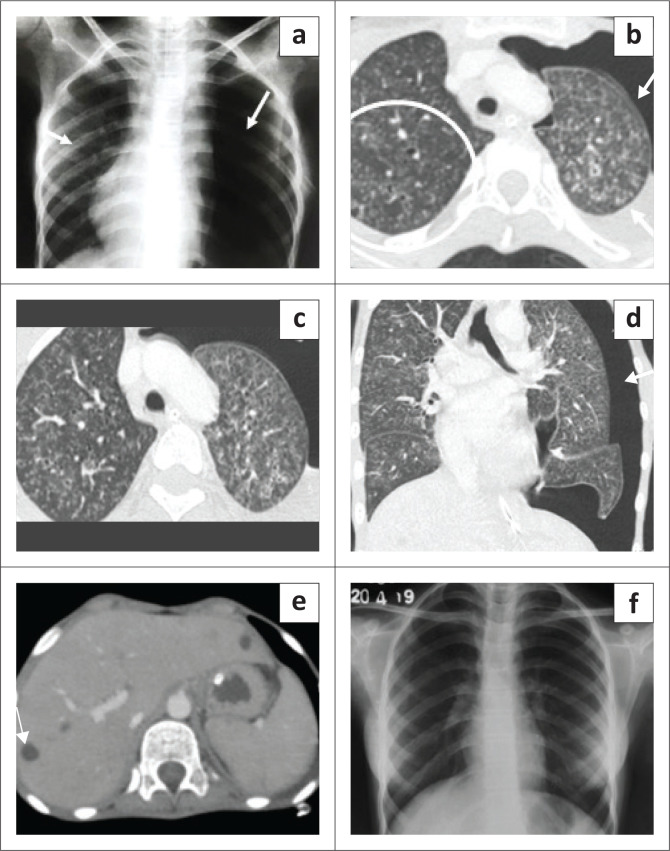
A 13-year-old lean, underweight female with disseminated tuberculosis. Chest radiography (CXR): (a) shows a left pneumothorax (long arrow) with the infiltrates in the right mid zone (short arrow). Contrast-enhanced CT chest axial (b, c) and coronal (d) images reveal a left hydropneumothorax (white arrow) with underlying collapse, multiple bilateral centrilobular nodules showing tree-in-bud opacities and a few tiny lung cysts (circle), better seen on the zoomed-up image (c). Axial section of the abdomen (e) reveals a few liver granulomas (arrow). (f) Follow-up CXR performed after 5 months appeared normal.

A 13-year-old emaciated, underweight girl presented with a cough for 12 days and projectile vomiting, associated with altered sensorium, blurring of vision and double vision over the preceding 4 days. There was a history of low-grade fever for 1 month associated with decreased appetite, weight loss and night sweats. The patient also had a positive contact history for pulmonary TB from her grandmother. Non-contrast CT brain at another facility (not shown) revealed communicating hydrocephalus and a hypodense fluid collection in the prevertebral space at the C2–C3 vertebral level, suggestive of a prevertebral abscess. Clinical and cerebrospinal fluid (CSF) findings suggested the diagnosis of tuberculous meningitis and the child was started on ATT. During her hospital stay, she developed sudden respiratory distress. The CXR (Figure 5) revealed a left pneumothorax with a few infiltrates in the right mid zone. An intercostal drainage (ICD) tube was subsequently placed. Sputum GeneXpert detected MTB. A CT of the chest (Figure 5) demonstrated a left hydropneumothorax with underlying collapse, multiple centrilobular nodules and a TIB pattern in both lungs, a few tiny cysts in both upper lobes, patchy consolidation in the right lower lobe, mediastinal lymph nodes with calcification, and hepatosplenomegaly with multiple liver and splenic granulomas. The patient improved on ATT and follow-up CXR (Figure 5) after 5 months revealed resolution of the radiographic findings.

### Case 4

**FIGURE 6:**
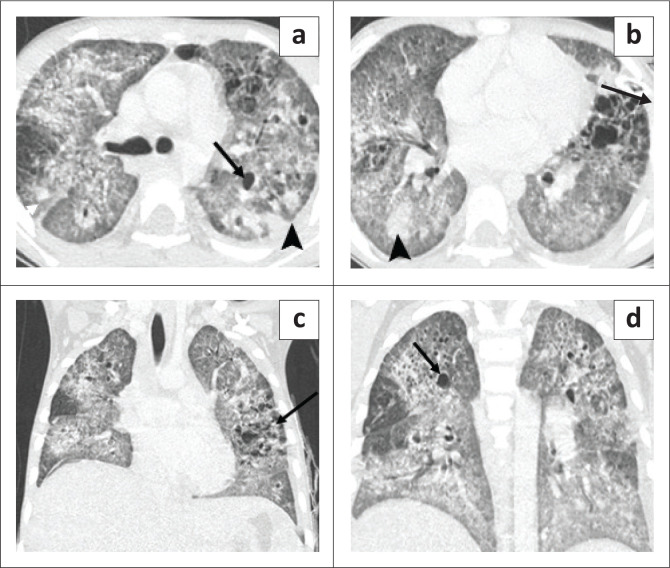
Contrast-enhanced CT chest axial (a, b) and coronal (c, d) images reveal extensive ground glass opacities and confluent nodular opacities in both lungs, along with patchy consolidation in both lower lobes (arrowhead), small lung cysts (arrows), pulmonary interstitial emphysema in the left upper lobe (long arrow in c). Left-sided intercostal drainage is in situ (arrow in b).

A 7-year-old girl was admitted with complaints of fever over 3 weeks, tachypnoea and cough for 4 days. In view of the respiratory distress, the child was transferred to the paediatric intensive care unit on day 5 post-admission. Mycobacterium tuberculosis was detected on sputum GeneXpert evaluation, and ATT was initiated. On day 11, the child experienced a sudden exacerbation of her respiratory distress. The CXR (not shown) revealed a left pneumothorax, for which an ICD was inserted. Subsequent CECT chest (Figure 6) revealed extensive GGOs and confluent nodular opacities in both lungs with patchy consolidation in both lower lobes, along with small cysts and bronchiolar dilatation. Other CT findings were mediastinal lymphadenopathy, pulmonary interstitial emphysema in the left upper lobe, minimal left pneumothorax with a pleural effusion, hepatosplenomegaly and hypodense non-calcified granulomas in the liver and spleen. The spleen also demonstrated a few small wedge-shaped regions of non-enhancing parenchyma suggestive of old infarcts. The patient responded well to ATT and recovered.

### Case 5

**FIGURE 7:**
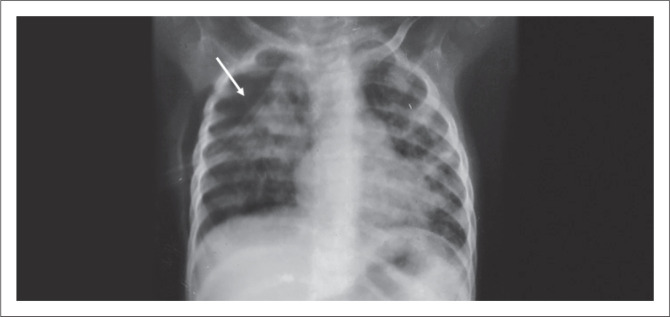
An 18-month-old male, follow-up case of disseminated multidrug resistant (MDR) TB on MDR regime. Chest radiography shows multiple cystic lucencies, ill-defined opacities in both lungs and a right pneumothorax with an intercostal drainage tube in situ (arrow).

**FIGURE 8:**
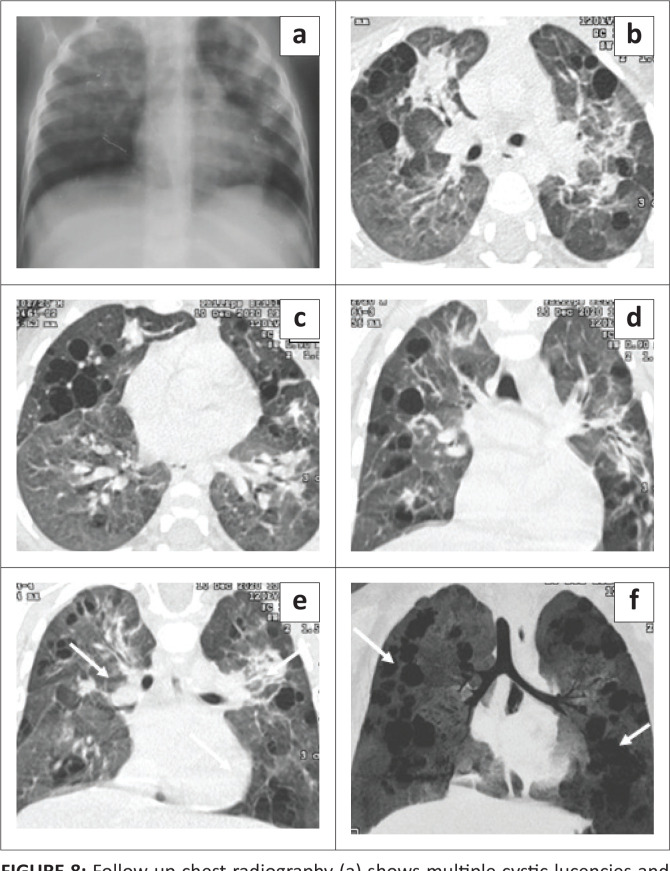
Follow-up chest radiography (a) shows multiple cystic lucencies and ill-defined opacities in both lungs, predominantly in the upper zones. Contrast-enhanced CT chest axial (b, c) and coronal (d, e) images reveal resolving consolidation with associated architectural distortion (arrows in e), traction bronchiectasis in the bilateral perihilar regions and multiple cysts of varying sizes in both lungs (arrows in f), better appreciated on the minimum intensity projection (MinIP) image.

An 18-month-old male with disseminated MDR TB, on an MDR regimen for 5 months, with severe acute malnutrition and recurrent pneumothoraces was being followed up. The patient had a history of a recent admission with a right-sided pneumothorax (Figure 7) and pneumonia. He was managed with ICD tube drainage and IV antibiotics and subsequently discharged. A follow-up CXR (Figure 8) after 2 months showed multiple cystic lucencies and haziness in both lungs, predominantly in the upper zones. The CT chest (Figure 8) revealed resolving consolidation with associated architectural distortion and traction bronchiectasis in the perihilar regions along with multiple cysts of varying sizes in both lungs. The patient responded to ATT (MDR regimen) and subsequently recovered.

## Discussion

A variety of lung diseases can cause or mimic cysts in the lung in the paediatric population. Cystic lung disease can occur in a myriad of conditions, which can be congenital as well as acquired, such as bronchogenic cysts, intralobar pulmonary sequestrations, cystic adenomatoid malformation of the lung, tracheobronchial papillomatosis, Langerhans cell histiocytosis, lymphocytic interstitial pneumonia, post-infectious states (*Pneumocystis jiroveci*) and many more. Cyst-like lesions or mimics include cystic bronchiectasis, cavities, pneumatoceles and honeycombing.^2,3,4^ Tuberculosis as a cause of multiple thin-walled lung cysts in children is rare. To the best of our knowledge, there are only a few case reports on CT findings that describe cystic lung changes in children with tuberculosis.^1,4,5^

Unusual CT findings for pulmonary TB are more common in a subset of patients, especially immunocompromised patients, malnourished patients, those with autoimmune deficiency syndromes, the elderly, alcoholics and those with diabetes mellitus.^6^ Among the presented patients, all had varying degrees of malnutrition, but none of them were infected with HIV.

Various pathological mechanisms are postulated to be responsible for the development of cystic lesions in tuberculosis. Cyst formation may be the result of granulomatous involvement of the bronchioles with a resultant check-valve mechanism caused by mural inflammation, oedematous luminal narrowing of the bronchioles by caseous material and peribronchiolar fibrosis.^1,2,3,4,5,6,7,8,9^ Scarring of the larger proximal bronchi with distal dilatation and interstitial air leakage with tuberculoma rupture have also been implicated in the pathogenesis.^1,3,4,5,6,7^ Other potential mechanisms include healed tubercular cavities re-lined by epithelia, cystic bronchiectasis and cysts developing post-isoniazid treatment in some cases.^1,4,7^

Most patients with tuberculosis who develop lung cysts have extensive bilateral infiltrative and exudative disease as a result of the pneumonic process.^1,4,7^ All the described cases had disseminated TB (with involvement of two or more noncontiguous sites), along with diffuse, bilateral lung involvement. These cystic lung lesions are often associated with centrilobular nodules and branching opacities in surrounding areas.^2,3,6^ Antibiotics were administered in all five cases, and none showed superadded bacterial infection. Three cases revealed numerous nodules, GGOs and consolidations. In Case 2, two CT studies were performed 2 months apart, which demonstrated a reduction in the lung nodules on treatment, with a corresponding increase in the number of cysts. Two cases showed predominantly cystic changes, with only a few nodules. One of these (Case 5) had received 5 months of ATT for MDR TB. In the presented cases, the cystic lesions developed during isoniazid treatment, rather than before or after treatment. This is in contrast to previous literature that describes certain instances of patients developing cystic lung lesions after isoniazid treatment.^7^

Tuberculous cysts are prone to rupture, leading to the development of recurrent pneumothoraces or pneumomediastinum.^1,2,7,9^ Pneumothorax was seen in four of the cases, likely as a result of cyst rupture. However, in Case 1, both cyst rupture and additional mechanical ventilation possibly played a role in the development of pulmonary interstitial emphysema and pneumomediastinum, which is difficult to differentiate radiologically.

According to previous literature, the evolution of cysts in tuberculosis may show varying severity, extent and an unpredictable outcome during the course of the disease. In some cases, cysts are reversible, while in others, the cysts remain static without progression and may persist on follow-up imaging.^1,2,3,7^ In this study, the cystic lesions were not evident on follow-up CXR in two cases, while one case (Case 5) showed persistent linear and fibrotic opacities on follow-up.

## Conclusion

Pulmonary tuberculosis with multiple cysts should be differentiated from other cystic lung diseases. Cystic changes are among the atypical presentations of TB and should be considered as a possible cause of acquired cystic lung disease in appropriate clinical settings, especially in endemic regions. These cases require prompt management to avoid lifelong complications such as those associated with recurrent episodes of pneumothorax necessitating multiple chest tube insertions, hospitalisations and the development of irreversible fibrotic lung changes.
